# Referral for Cardiac Amyloidosis in Patients Who Underwent Transcatheter Aortic Valve Replacement: Results of the Quality Outcome Project

**DOI:** 10.7759/cureus.45024

**Published:** 2023-09-11

**Authors:** Dae Hyun Lee, Gerry S Eichelberger, Vandan Patel, Ronak Chhaya, Arjun Khadilkar, Jennifer Bishop, Hiram Bezerra, Guilherme Oliveira, Fadi Matar, Bibhu D Mohanty, Joel Fernandez

**Affiliations:** 1 Department of Cardiovascular Medicine, University of South Florida, Tampa, USA; 2 Department of Internal Medicine, University of South Florida, Tampa, USA

**Keywords:** quality improvement research, transthyretin cardiac amyloidosis, attr cardiac amyloidosis, transthyretin amyloidosis, transcutaneous aortic valve replacement, quality improvement projects, cardiac amyloid, aortic stenosis

## Abstract

Background

Transthyretin cardiac amyloidosis (ATTR) is an important comorbidity present in severe aortic stenosis (AS). The purpose of this study was to raise awareness of ATTR in patients who underwent transcatheter aortic valve replacement (TAVR) for severe AS among healthcare providers and patients.

Methodology

We reviewed 197 consecutive TAVR cases performed from 2019 to 2020. Adapting predefined high-risk features for ATTR based on prior literature, we contacted the patients to discuss our clinical suspicion of ATTR and offered a referral to a cardiac amyloid specialist.

Results

We identified 125 (69.4%) patients who had high-risk features of ATTR. Of the 105 patients contacted, 44 patients agreed to referral, 46 patients were not able to be contacted after several attempts, and 15 patients declined referral. Of the 44 patients who agreed to the referral, 20 patients completed the evaluation for cardiac amyloidosis, all of whom were negative for transthyretin and light-chain cardiac amyloidosis.

Conclusions

Our attempt to detect ATTR in prior TAVR patients was unsuccessful two to three years post-TAVR. We believe that early detection of cardiac amyloidosis close to the timing of TAVR is important and the most effective means.

## Introduction

Aortic stenosis (AS) is a common valvular disorder mostly affecting the elderly population [1]. It is caused by degenerative calcium deposition of the aortic valve. AS presents as dyspnea, chest pain, syncope, and congestive heart failure symptoms due to pressure overload leading to left ventricle (LV) remodeling and impairment of systolic or diastolic function [1]. A relatively common disease entity that occurs in AS is cardiac amyloidosis (CA). CA has two major subtypes, namely, transthyretin cardiac amyloidosis (ATTR) and light-chain amyloidosis. Multiple prospective studies have shown that CA is quite common, with about 15-20% of patients with AS having concomitant ATTR [2]. The pathophysiology of CA in AS is unclear but thought to be related to high stress/inflammation. The treatment for severe AS is surgical or transcatheter aortic valve replacement (TAVR), which improves survival [3]. Concomitant CA and AS have worse outcomes than AS without CA in meta-analysis and leading amyloidosis centers [4].

Both severe AS and CA can lead to heart failure. Moreover, severe AS and CA have some common features on echocardiogram, including cardiac hypertrophy and diastolic dysfunction. However, CA has characteristic apical sparing of strain on echocardiogram [5]. In addition, the discrepancy between the degree of the voltage of QRS and echocardiographic measurement of wall thickness (EKG-echocardiogram discrepancy) can be assessed through the voltage mass ratio, which is low in patients with CA [6]. The diagnosis of CA is through either an endomyocardial biopsy or a 99m technetium pyrophosphate (99mTc-PYP) scan. The recent landmark ATTR-ACT trial showed that the use of tafamidis in ATTR had a reduction in all-cause mortality and cardiovascular-related hospitalization/quality of life and functional capacity [7]. With the advent of novel therapeutic options with improved survival, the screening of CA became more clinically meaningful, as shown by recent studies investigating the presence of ATTR in carpal tunnel syndrome and AS [8].

There is a gap in care and underrecognition of ATTR-CA in patients who already underwent TAVR, as there was a paucity of knowledge before 2019 on the risk of ATTR in the setting of AS. Therefore, this study aimed to identify patients with a high risk for the presence of CA in patients who underwent TAVR in our institution and to refer them to a heart failure cardiologist for further evaluation and screening for CA.

This article was previously posted to the medRxiv preprint server on April 27, 2020. The preliminary results of this article were previously presented as a meeting abstract at the 2021 American Heart Association Scientific Session on November 14, 2021, and the 2022 American College of Cardiology Scientific Session on April 3, 2022.

## Materials and methods

This study was reviewed by the University of South Florida Institutional Review Board and was approved as a quality improvement project (STUDY000938). This study is a single academic center quality improvement project involving patients diagnosed with severe AS who underwent TAVR. There was no concern for ethical issues by implementing standard clinical care and practice to adhere to all federal and institutional regulations.

Based on prior literature and guidelines for diagnostic testing for CA, we selected predefined risk factors for the presence of CA (Table 1).

**Table 1:** Clinical risk factors for the presence of cardiac amyloidosis in aortic stenosis.

Risk factors	Present in high-risk patients (N = 125)
Increased wall thickness on echocardiogram (PWD, IVS >12 mm)	81 (64.8%)
Low voltage on limb leads of 12-lead EKG	20 (16.0%)
Low Sokolow-Lyon index <2.0 mV	64 (51.2%)
Low voltage mass ratio <1.5	44 (35.2%)
Diastolic dysfunction grade 2 or above	64 (51.2%)
Low-flow low-gradient aortic stenosis	6 (4.8%)
Atrial arrhythmia or right or left bundle branch block	75 (60.0%)
Systemic syndrome of amyloid	9 (7.2%)

We retrospectively reviewed the electronic medical records of patients who underwent TAVR at our institution from January 2019 to December 2020 (a total of 197 patients). Patients were designated high-risk group for the presence of CA if they had two or more of these risk factors. After a discussion with the structural cardiologists, a referral was made to an advanced heart failure cardiologist. Patients were contacted via phone to inform them of the quality improvement initiative and the purpose and plan for referral to a heart failure cardiologist. Heart failure cardiologists then saw patients who agreed to the referral. After evaluation by the heart failure cardiologist, if a patient was deemed clinically appropriate for screening for CA, a 99mTc-PYP scan was performed, along with screening for plasma cell dyscrasia through serum and urine electrophoresis, immunofluorescence, and free light-chain quantification.

The primary outcome of the quality improvement project was the diagnosis of CA in patients who underwent TAVR. We compared the number of patients who underwent diagnostic testing for CA before our intervention (active screening for risk factors for CA) based on interventional cardiologists’ standard of care and after our intervention.

All data are presented as numbers (percentage, %). Data were collected and managed using the REDCap electronic data management system hosted at the University of South Florida [9]. All statistical analyses were performed through R (version 4.0.4) [10].

## Results

**Figure 1:**
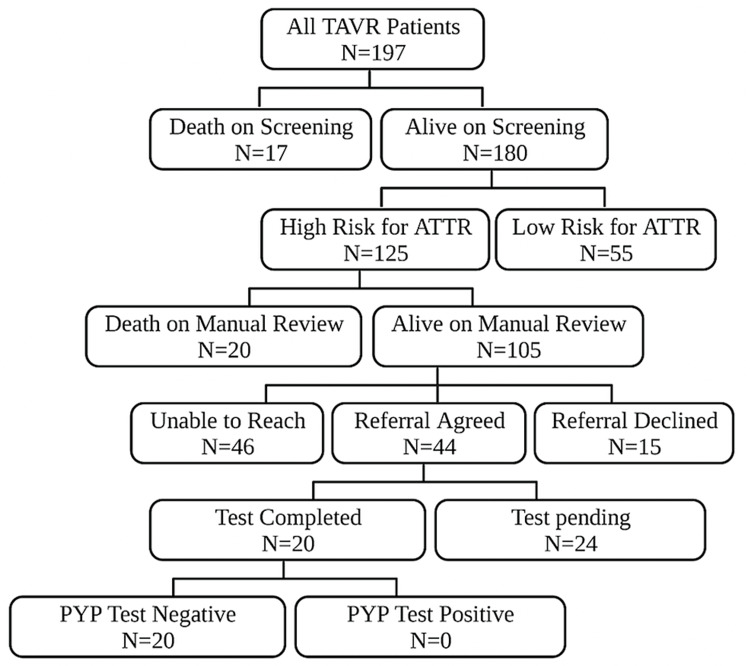
Patients who underwent TAVR for severe AS from January 2019 to December 2020. Study methodology algorithm. A total of 197 patients underwent TAVR from 2019 to 2020 at our institution. There were 37 patients who were deceased on chart review. There were 105 patients who had the presence of high-risk features for ATTR. We attempted to contact these patients but were unable to contact 46 patients after multiple attempts. Of the 59 patients we were able to contact, 44 patients agreed to the referral to an advanced heart failure cardiologist. Twenty patients completed diagnostic tests; all were negative for ATTR or AL amyloidosis. AS: aortic stenosis; ATTR: transthyretin cardiac amyloidosis; TAVR: transcatheter aortic valve replacement. Figure created using BioRender.com.

A total of 197 patients underwent TAVR for severe AS from January 2019 until December 2020 (Figure 1).

A total of 17 patients were deceased on the initial chart review. Of the patients who were alive on screening (N = 180), 125 (69.4%) patients had the high-risk criteria for the presence of ATTR and an indication for referral to a heart failure cardiologist for evaluation of CA.

In patients who met the high-risk criteria for the presence of ATTR, the most common risk factors were increased wall thickness on echocardiogram (64.8%), arrhythmias or right/left bundle branch block (60.0%), diastolic dysfunction grade 2 or higher, and low Sokolow-Lyon index (51.2%), as summarized in Table 1.

We made attempts to contact the patients and found an additional 20 patients who were deceased after the contact attempt. Of the 105 living patients, 44 patients agreed to a referral to a heart failure cardiologist, 15 patients declined the referral, and 46 patients were unable to be contacted despite three separate phone call attempts. Complete diagnostic evaluations were performed on 20 patients. However, none of the patients had a new diagnosis of CA. There are 24 patients pending complete evaluation.

## Discussion

We describe our quality improvement project on the referral of patients who underwent TAVR for evaluation of CA. Recently, multiple studies have shown that the incidence of concomitant ATTR with severe AS is high at 15-20% [2,11,12]. The diagnosis of ATTR gained interest with the therapeutic development of tafamidis with meaningful survival benefits. After such research development, there was an increased awareness of the detection of ATTR in patients with severe AS. However, patients who have undergone TAVR in the recent past, before widespread knowledge of the relationship between ATTR and AS, are at risk for lack of evaluation for ATTR. Accordingly, our cohort of patients who underwent TAVR from 2019 to 2020 did not undergo a 99mTc-PYP scan peri-procedurally.

We routinely perform cardiac workups for TAVR evaluation, including cardiac and non-cardiac history, EKG, and echocardiogram. We have utilized well-established risk factors that would now allow us to suspect the presence of CA, including low voltage on limb leads of EKG, low Sokolow-Lyon index on EKG, left ventricular hypertrophy on echocardiogram, low voltage mass index (EKG/echo discrepancy), grade 2 or higher diastolic dysfunction, presence of arrhythmia or AV/bundle branch block, low-flow low-gradient AS, systemic syndrome of ATTR (carpal tunnel syndrome, lumbar stenosis, rotator cuff injury) [13-16]. Unfortunately, at the time of our study, strain analysis was not available routinely, and we could not include the apical sparing pattern as part of our criteria. A significant number of patients (over 60%) met the high-risk criteria for the presence of CA. We initially expected that ATTR would be present in 10-15% of all patients who underwent TAVR, consistent with the literature. However, we could not identify any patients with ATTR diagnosed via the PYP scan.

There are several explanations for the lack of diagnosis of CA in our cohort. First, there were a significant number of patients whom we were not able to contact by telephone despite several attempts. To fully assess the survival of the patients we were unable to reach, we manually performed a Google search of the patient’s name and date of birth for the obituary. Second, there was a significant number of patients who were referred to a heart failure clinic but not seen in the clinic, or an evaluation for CA was not performed. One of the most challenging factors was the COVID-19 pandemic. This made it difficult to schedule the patients in a timely fashion. Many patients were lost to long-term follow-up since TAVR (maximum two to three years ago at the time of call) and the nature of the tertiary referral center for TAVR. Third, the selection of high-risk features of CA may not be perfect. However, we have utilized existing literature and guidelines to decide on referral. The number of patients at high risk was much higher than the known incidence of concomitant CA and severe AS; thus, we hypothesize that all ATTR-AS patients were included in our high-risk patients. In that case, the ATTR-AS patients would be in the group of (1) referred but not seen, (2) deceased, and (3) unable to be contacted via phone. We cannot ascertain what the reason is. Lastly, a similar effort was made in patients with atrial fibrillation [17]. In the first cohort of over 800 patients with atrial fibrillation, 11.9% met the criteria for high-risk features of ATTR, defined as age above 60 and LV wall thickness of 12 mm or above. However, only one patient was diagnosed with ATTR. In the second cohort of patients with over 800 patients with atrial fibrillation with high-risk features with some modification (age above 60 and LV wall thickness of 15 mm or above), no patient was diagnosed with ATTR. Another limitation of our study is that we were not able to obtain the cause of death in all patients, given the retrospective nature of our study. We did not collect information regarding postmortem examination, which would have detected CA during autopsy.

Despite our efforts in attempting to identify patients with concomitant CA and AS, we were unable to identify any patients. We believe that the best screening method for CA would be at the time of evaluation for TAVR. These patients are older and may have difficulty coming to the clinic for evaluation for CA. Therefore, evaluation for CA before TAVR, during pre-TAVR evaluation, or during follow-up outpatient visits after TAVR may be a good time. This may be especially the case if patients have continued heart failure-like symptoms after TAVR. Further studies evaluating the screening of all or a subset of patients for CA should be conducted, considering the cost-effectiveness and the risk of extra radiation exposure from a PYP scan.

## Conclusions

We describe our quality improvement project for proper referral for the evaluation of ATTR in patients who underwent TAVR. Our quality improvement project for the referral of patients who underwent TAVR for the evaluation of ATTR was not successful in diagnosing CA two to three years after TAVR. While we were not able to identify any patients based on our algorithm, we were able to extrapolate several meaningful results that can be used going forward as new knowledge of ATTR amyloidosis surfaces. This project highlights the ongoing importance of early detection of ATTR amyloidosis and the significance of identifying the diagnosis early, as treatment options are now available and accessible. We believe that the early detection of CA close to the timing of TAVR is of importance and the most effective means. Going forth, ideally, screening all patients with AS for amyloidosis would be of suggested benefit. It is likely that performing an amyloidosis workup at the onset of consideration for TAVR for AS patients would be useful and have more profound results with better follow-up of patients.
