# Correction: Referral for Cardiac Amyloidosis in Patients Who Underwent Transcatheter Aortic Valve Replacement: Results of the Quality Outcome Project

**DOI:** 10.7759/cureus.c141

**Published:** 2023-10-31

**Authors:** Dae Hyun Lee, Gerry S Eichelberger, Vandan Patel, Ronak Chhaya, Arjun Khadilkar, Jennifer Bishop, Hiram Bezerra, Guilherme Oliveira, Fadi Matar, Bibhu D Mohanty, Joel Fernandez

**Affiliations:** 1 Department of Cardiovascular Medicine, University of South Florida, Tampa, USA; 2 Department of Internal Medicine, University of South Florida, Tampa, USA

The author list of this article has been corrected to include Dr. Bibhu D. Mohanty, who was mistakenly omitted by the authors. This omission was not discovered until after publication. The authors regret the error. Dr. Bibhu D. Mohanty is now listed as the 10th author.

